# Prevalence and Occlusal Risk Factors for Fractured Incisors among 11–12-Year-Old Children in the Trinidad and Tobago Population

**DOI:** 10.3390/dj8010025

**Published:** 2020-03-06

**Authors:** Trudee Hoyte, Anne Kowlessar, Anil Ali, David Bearn

**Affiliations:** 1 School of Dentistry, Faculty of Medical Sciences, University of the West Indies, St. Augustine, Trinidad; annekowlessar@gmail.com (A.K.); anilali66@hotmail.com (A.A.); 2 School of Dentistry, University of Dundee, Scotland DD1 4HN, UK; dbearn@gmail.com

**Keywords:** cross-sectional survey, prevalence, occlusal risk factors, fractured incisors, Trinidad and Tobago

## Abstract

This cross-sectional survey was carried out to ascertain the prevalence of fractured incisors in 11–12-year-olds. In addition we explored the relationship with overjet, incompetent lips, incisor inclination and to determine if there was any association with ethnicity and gender. All permanent incisors were examined in 672 children comprising 356 females (53.1%) and 315 males (46.9%). The sample comprised 11–12-year-olds in high schools across Trinidad and Tobago. Statistical analysis was undertaken using Chi-square test, independent t-test, and binary logistic regression. The prevalence of fractured incisors was 18.9%. Boys presented with an increased incidence of fractured incisors than girls. 86.3% of dental trauma was untreated. The average overjet of subjects with fractured incisors was 4.2 mm. 18.62% of subjects with fractured incisors had incompetent lips. The most common malocclusion (18.81%) with fractured incisors was class 2 division 1. The Afro-Trinidadian ethnicity had the highest prevalence of fractured incisors (11.0%) when compared to mixed ethnicities, which was statistically significant. Maxillary central incisors were the most commonly injured teeth. Most patients delayed in seeking dental treatment for fractured incisors in our population. Early orthodontic treatment is recommended to help reduce the risk of dental trauma.

## 1. Introduction

Oral injuries are the fourth most common area of bodily injuries among 7–30-year-old individuals [1]. Dental trauma (traumatic dental injury) results from an impact to the teeth and/or other hard and soft tissues within and around the vicinity of the mouth and oral cavity [2]. These injuries are common in certain groups, no individual is ever at zero risk through their activities of daily living [2].

It is a serious condition among young children as dental injuries result in aesthetic, and functional problems involving the maxilla and mandible. Dental injuries can also cause psychological disturbances for the child, parent and the dentist.

Dental trauma presents as a public health problem and in some countries where caries have decreased, it can be considered the major risk to the anterior teeth [3,4].

The expense to the injured person and the community throughout the world has been substantial in terms of time and cost [2,5,6]. The average number of visits during one year due to sustaining dental trauma ranges from 1.9 to 9.1 [7]. It has also been discovered in Australia that only one-third of the patients presented for dental treatment within 24 hours of the injury, while the remainder delayed seeking treatment for varying times up to 1 year [8].

Trinidad and Tobago is a cosmopolitan country where according to the central statistical office, the three main ethnic groups are Afro-Trinidadian, Indo-Trinidadian, and mixed ethnicity. Bimaxillary proclination is the most prevalent malocclusion found in 68.8% of the population [9]. Clinical features of bimaxillary proclination include incompetent lips and an increase in overjet. In most societies, these features have been identified as risk factors for trauma [10,11].

Most studies however, have examined the relationship between incisor fracture and single features like sex, age, and overjet using univariate statistical methods.

The hypotheses for this cross-sectional study are

1. There is a high prevalence of fractured incisors in the Trinidad and Tobago society.
2. Fractured incisors prevalence is not equal in all ethnic groups in Trinidad and Tobago.
3. The is no gender predilection with fractured incisors in Trinidad and Tobago.
4. There is a high prevalence of occlusal risk factors for fractured incisors in Trinidad and Tobago.

Currently, there is little epidemiological data on dental trauma in Trinidad and Tobago. The aim of this study was to firstly, investigate the prevalence and occlusal risk factors for dental trauma in high school children in Trinidad and Tobago. Also, to asses any association with ethnicity and gender.

## 2. Patients and Methods

A cross-sectional survey was carried out on 672 high school children aged 11 to 12 years old in 141 public schools in the twin island republic of Trinidad and Tobago. These schools were located across the twin island republic representing both rural and urban populations during the period June 2013 to April 2016. This study is reported in accordance with STROBE guidelines.

The ethics committee of The University of The West Indies granted approval for this cross-sectional survey in April 2013. The Trinidad and Tobago Ministry of Education gave approval for the research to be conducted in high schools across the country. A letter was sent to school Principals requesting permission to conduct the research. Another letter was sent to parents asking for permission for their children’s participation. Only students from whom consent was obtained from both parents and child were examined.

For the purpose of determining the adequacy of the sample size, the Chi Square analysis with fractured incisors and lip competence was treated as the main analysis. Using the G* Power (Fau et al. 2007) [12] it was calculated that a 2 × 2 Chi Square with 1 degree of freedom and our sample size of 672 achieved a power (to 7 decimal places) of 100% to detect medium effect sizes (w = 0.3) and 73.4% power to detect small effects (w = 0.1). Accordingly, this study was more than adequately powered to detect all but the smallest effects.

Dental examination was carried out by a single dentist (TH) supported by a recorder. The students were seated on a chair in a well-lit area. Traumatic injuries to the incisors were recorded. Students who had already undergone previous orthodontic treatment were excluded from this cross-sectional survey so that orthodontic treatment for an unknown reason and as a confounding factor was removed.

The following data were recorded

- Patient demographics: Information included age, sex, ethnicity
- Trauma History: Trauma was recorded when there was
  ○ Fracture involving enamel
  ○ Fracture involving enamel and dentine
  ○ Fracture involving enamel and dentine and pulp
  ○ Discoloration of the crown as a result of traumatic injury (verified by an interview)
  ○ Presence of a restoration done on a tooth as a result of traumatic injury (verified by an interview)
- Skeletal Relationships: The patients were assessed in profile view into Class1, Class 11, Class 111.
- Morphologic malocclusion: The following were assessed with the subjects in centric occlusion.

1. Overjet was measured with a millimeter ruler as from the incisal edge of the most labial maxillary central incisor to the most labial mandibular central incisor distance to the occlusal plane.
2. Lip competence was evaluated with the lips in rest position and scored as competent once there was no strain. If lip strain was evident on closure the lips were scored as incompetent.
3. Assessment of malocclusion was done with teeth in centric occlusion, the relationship between the upper and lower incisors were assessed (British Standards Institute 1983).

## 3. Statistical Analysis

The data was analyzed using IBM SPSS Statistics for Windows version 22 (IBM Corp., Armonk, NY, USA).

Descriptive analysis was undertaken and statistical associations for dental injuries with sex, ethnicity, incisal overjet, lip competence and skeletal pattern were calculated using Chi-square test and independent t-test. These analyses were used to test associations between occurrence of occlusal features and dental trauma. Binary logistic regression was then performed to estimate the predictive value of ethnicity, overjet and lip competence for the probability of incisor injury.

## 4. Results

A total of 672 children across high schools in Trinidad and Tobago aged 11–12 participated in this cross-sectional survey. The overall prevalence of fractured incisors was 18.9%. Fracture of the upper incisors showed a prevalence of 18.5% and lower incisors 0.4%. Among the children who had experienced traumatic dental injuries to the teeth 86.3% of children had untreated fractured incisors. There were more girls (n = 356, 53.1%) than boys (n = 315, 46.9%). Boys (9.52%) experienced more fractured incisors than girls (7.58%) however, this difference was not statistically significant, p > 0.05.

Afro–Trinidadian ethnicity had the highest prevalence of fractures at 11.0%, the Indo-Trinidadian ethnicity had a prevalence of 6.19% and the Mixed ethnicity had the lowest prevalence, 5.93%. The differences in prevalence associated with ethnicity was however not statistically significant (p = 0.15).

18.62% of subjects with incompetent lips had fractured upper incisors compared with 8.54% with competent lips. This difference was statistically significant p = 0.001 (Table 1). The mean overjet of subjects with fractured incisors was 4.2 mm ± 2.1. The mean overjet of subjects in the non-fractured incisors group was 3.48 mm ± 2.01. An independent sample t-test for equality of means showed the difference with overjet between fractured and sound incisors was statistically significant p = 0.03.

Children with a class 2 division 1 incisor relationship were more likely to have a fractured incisor compared with other malocclusions (Table 2). The difference was statistically significant p = 0.021.

Binary Logistic regression was suggestive of a relationship between fractured incisors and mean overjet, ethnicity (Afro-Trinidadian and Mixed ethnicity Trinidadian), and incompetent lips. The above were shown to be predictors of fractured incisors. The odds ratio showed that as the overjet increased the chances of a fractured incisor increased. This association was statistically significant, p = 0.004. In terms of ethnicity, moving from Afro-Trinidadian to Indo-Trinidadian the increase of fractured incisors was not statistically significant, p = 0.59. Comparing Afro-Trinidadians to Mixed ethnicity Trinidadians the difference in fractured incisors was however statistically significant, p = 0.035. Moving from competent to incompetent lips the odds of a fractured incisor increased and this was statistically significant, p = 0.02 (Table 3). Mean overjet, incompetent lips, and moving from Afro-Trinidadian to Mixed ethnicity Trinidadian were all statistically significant, p < 0.05. This suggests that you can make an educated guess if a subject is susceptible to incisal fracture based on these three parameters.

The 95% confidence interval showed that mean overjet and incompetent lips are the more significant predictors of dental trauma. The classification table showed that fractured incisors would be mis-classified in nearly all but 1.6% fractured incisor patients (Table 4).

## 5. Discussion

The prevalence of fractured incisors in 11–12-year-old school children in Trinidad and Tobago was 18.9%. This is comparable to another study in the Caribbean on Dominican school children which found a prevalence of 18.1% [13] and in the United States which found a prevalence of 18% [14]. In other studies, the reported prevalence rates varied from 4.1% in Malaysian children [15] to 19.8% in Finish children [16]. Differences in sampling techniques and application of diagnostic criteria could be responsible for the varying prevalence rates among studies [17]. This study confirms findings in other studies that the maxillary incisors were more often affected with traumatic injuries than mandibular incisors and maxillary central incisors were affected more than lateral incisors [14,18,19,20].

This is possibly due to the maxillary central incisors having a prominent position in the arch, this is in agreement with several studies [13,15,16,21,22]. Most of the children in this study did not seek treatment, 86.3% of the fractures were unrepaired. This confirms findings by other studies [13,23]. There are several possible reasons why a patient would delay in seeking treatment. This can be explained by the lack of pain or any symptoms, patients giving a low priority to their dental injuries, unavailability of dentist due to travel, sickness, or other commitments, long wait periods at the dental hospital and patients being unaware of dentist after hours service [8].

In this study, boys were affected by fractured incisors more than girls and this confirms the findings of numerous studies [14,15,16,24,25,26] but the difference was not statistically significant. Noteworthy is the Dominican study which reported higher levels in girls but this difference was not statistically significant [13]. One explanation for both results is the increased participation of girls in risk activities and sports [10].

In this study there was a higher prevalence of subjects of African descent with fractured incisors as in other studies [14,19]. However, the difference was not statistically significant.

The reported predisposing factors for dental trauma include an increased overjet, protrusion of upper incisors, lip incompetence, inadequate lip coverage and accidental proneness [1,10,24]. This study found 37.5% of patients with fractured incisors had incompetent lips which was significantly more than reported by other authors [27]. This confirms the opinion that persons with incompetent lips are more likely to injure their incisors [21].

Class 2 division 1 incisors where the upper incisors are protrusive were found to be more likely to have a fractured incisor compared to other malocclusions, also children with a mean overjet of 4mm and above were found to have a higher prevalence of fractured incisors. This study confirms the findings in other studies that children with protrusive incisors and an increase in overjet have a higher incidence of trauma [10,21,22].

The binary logistic regression model showed that mean overjet and incompetent lips had a clear association with fractured incisors but the predictive value was low.

Dearing [11] stated that children with an overjet greater than 6mm should receive prophylactic orthodontic treatment. Two methods of prevention of dental trauma are available, wearing of mouthguards and orthodontic treatment. Early orthodontic treatment before age 11 has been recommended to prevent dental trauma [10,20,28,29]. The benefits of early class 2 division 1 treatment have been documented in randomized clinical trials [29]. Noteworthy is that growth modification was the objective of this early treatment and with the growth modification there was a decrease in overjet. This early reduction in overjet greatly reduces the cost to public health care for dental trauma. Koroluk [29] reported 29.1% of patients at the start of his randomized clinical trial (before age 9) had already had incisor trauma. He asserted shortly after incisor eruption overjet reduction should begin. Other prevention techniques that can be undertaken by patients include wearing of mouthguards, seatbelts, protective gear and participation in oral health promotion [8].

Understanding the epidemiology of dental trauma in Trinidad and Tobago requires more local studies. Oral health programs should include education on the need to seek immediate treatment.

## 6. Conclusions

- Males presented with more dental injuries than females but this was not statistically significant
- Differences in prevalence with ethnicity were only significant when Afro-Trinidadian and mixed ethnic groups were compared
- The most common injured tooth was the maxillary central incisor
- Increased overjet and incompetent lips, were clearly associated with incisor trauma but their predictive value was low.
- Use of mouthguards and early orthodontic treatment are recommended in these patients
- Many patients delayed seeking treatment for their injuries.

## Figures and Tables

**Table 1:** Relationship between fractured incisors and lip competence.

Lip Competence			Total
	Competence	Incompetence	
Fractured Incisors	45	27	72
Sound Incisors	482	118	600
Total	527	145	672
P = 0.01			

**Table 2:** Relationship between malocclusion and fractured incisor.

Incisor Relationship	Class1	Class 2 Division 1	Class2 Division 2	Class 3	Total
Fractured Incisor	33(10.54%)	19(18.81%)	0	20 (7.97%)	72
Sound	280	82	7	231	600
Total	313	101	7	251	672
P = 0.021					

**Table 3:** Binary Logistic Regression model containing the variables, mean overjet, ethnicity, lip competence.

Explanatory Variable	B	Relative Odds	95% Confidence Limits for Relative Odds	Significance
Mean overjet	0.137	1.147	1.044, 1.260	0.004
Afro-Trinidadian to Indo-Trinidadian	-0.124	0.883	0.565, 1.381	0.587
Afro-Trinidadian to Indo-Trinidadian	-0.640	0.527	0.291, 0.955	0.035
Lips	0.704	2.022	1.286, 3.180	0.02

**Table 4:** Classification Table showing predictive power of sample.

Observed	Predicted		
	Fracture		Percentage Correct
	No Fracture	Fracture	
No Fracture	542	3	99.4
Fracture	125	2	1.6
Overall Percentage			81.0
